# 

**Published:** 1899-01

**Authors:** 


					﻿Southern Medical Record
A Monthly Journal of Medicine and Surgery.
Vol. XXIX.
ATLANTA, GA., JANUARY, 1899.
No. 1?
BERNARD WOLFF, M. D., Editor.
Associa/rTEdkqbs :
A. W. GRIGGS, M. D.
j. McFadden gaston, Jr., m. d.
5 Mo^ADDEN <GASTON, M. D.
¡ .W*LÌjfe/p. WESTMORELAND, M. D
MICHAEL HOKH, M.D.?
TERMS: $1.00 Per Annum; Single Copy,/10Centsf^
'Conßnts on Page 3.
Entered at the Post-office at Atlanta. Ga.. as Second-class Matter.
				

